# Association between P2X7 Polymorphisms and Susceptibility to Tuberculosis: An Updated Meta-Analysis of Case-Control Studies

**DOI:** 10.3390/medicina55060298

**Published:** 2019-06-21

**Authors:** Mohsen Taheri, Hosna Sarani, Abdolkarim Moazeni-Roodi, Mohammad Naderi, Mohammad Hashemi

**Affiliations:** 1 Genetics of Non-communicable Disease Research Center, Zahedan University of Medical Sciences, Zahedan 9816743463, Iran; mohsen.taheri.gene@gmail.com; 2 Department of Genetics, School of Medicine, Zahedan University of Medical Sciences, Zahedan 9816743463, Iran; 3 Children and Adolescent Health Research Center, Resistant Tuberculosis Institute, Zahedan University of Medical Sciences, Zahedan 9816743111, Iran; hosna.sarani@gmail.com; 4 Department of Clinical Biochemistry, Iranshahr University of Medical Sciences, Iranshahr 9916643535, Iran; ak.moazeni@gmail.com; 5 Infectious Diseases and Tropical Medicine Research Center, Resistant Tuberculosis Institute, Zahedan University of Medical Sciences, Zahedan 9816743111, Iran; naderi597@gmail.com; 6 Department of Clinical Biochemistry, School of Medicine, Zahedan University of Medical Sciences, Zahedan 9816743175, Iran

**Keywords:** P2X7, polymorphism, tuberculosis, meta-analysis

## Abstract

*Background and Objectives:* Several studies inspected the impact of *P2X7* polymorphisms on individual susceptibility to tuberculosis (TB), but the findings are still controversial and inconclusive. To achieve a more precise estimation, we conducted a meta-analysis of all eligible studies on the association between *P2X7* polymorphisms and TB risk. *Materials and* *Methods:* Relevant studies were identified by searching the PubMed, Web of Science, Scopus and Google scholar databases up to November 2018. Twenty-four full-text articles were included in our meta-analysis. The strength of association between *P2X7* polymorphisms and TB risk was evaluated by odds ratios (ORs) and 95% confidence intervals (95% CIs) under five genetic models. *Results:* The findings of this meta-analysis revealed that the rs3751143 variant significantly increased the risk of TB in heterozygous codominant (OR = 1.44, 95%CI = 1.17–1.78, *p* = 0.0006, AC vs. AA), homozygous codominant (OR = 1.87, 95% CI = 1.40–2.49, *p* = 0.0004, CC vs. AA), dominant (OR = 1.50, 95% CI = 1.22–1.85, *p* = 0.0002, AC + CC vs. AA), recessive (OR = 1.61, 95% CI = 1.25–2.07, *p* = 0.001, CC vs. AC + AA), and allele (OR = 1.41, 95% CI = 1.19–1.67, *p* < 0.0001, C vs. A) genetic models. Stratified analysis showed that rs3751143 increased the risk of pulmonary tuberculosis (PTB) and extrapulmonary tuberculosis (EPTB) in all genetic models. Furthermore, the rs3751143 increased risk of TB in the Asian population. The findings did not support an association between the rs2393799, rs1718119, rs208294, rs7958311, and rs2230911 polymorphisms of *P2X7* and TB risk. *Conclusions:* The findings of this meta-analysis suggest that *P2X7* rs3751143 polymorphism may play a role in susceptibility to TB in the Asian population. More well-designed studies are required to elucidate the exact role of *P2X7* polymorphisms on TB development.

## 1. Introduction

Tuberculosis (TB) is a chronic infectious disease caused by the bacillus *Mycobacterium tuberculosis* (MTB). It remains a serious public global health problem. According to the World Health Organization (WHO) report, there were an estimated 10.4 million new cases of TB worldwide and approximately 1.3 million deaths in 2016 [1]. Approximately one-third of the general population is currently infected with Mtb, and nearly 5–10% of these infected individuals will progress to active TB [2,3]. Mounting evidence has proposed that host genetic factors play an important role in determining inter-individual difference in susceptibility to TB [4,5,6].

The human *P2X7* gene is mapped to chromosome 12 (12q24.31). It encodes cell-surface nucleotide receptors called P2X7 receptor (P2X7R) [7]. The P2X7R, a ligand-gated cation channel, is highly expressed on macrophages and other immune cells [8]. Activation of P2X7R by extracellular adenosine triphosphate (eATP) causes immediate opening of a cation selective channel, permitting the influx of Ca^2+^ and Na^+^ and the efflux of K^+^ [9]. In M. tuberculosis infection, activation of the P2X7R promotes a range of signaling cascades leading to the apoptosis of MTB-infected macrophages [10,11].

The *P2X7R* gene is indeed polymorphic. The precise correlation between the *P2X7* polymorphisms and susceptibility to TB is not completely documented. Several single nucleotide polymorphisms (SNPs) have been revealed that affect the function of this receptor which cause P2X7R loss-of-function (LOF) or gain-of-function (GOF) [8]. Many studies have inspected the association between *P2X7* polymorphisms and risk of tuberculosis in various populations, but the findings were inconsistent [12,13,14,15,16,17,18,19,20,21,22,23,24,25,26,27,28,29,30,31,32,33,34,35]. So we conducted an updated meta-analysis of all available eligible case-control studies published to date, focusing on the association between *P2X7* polymorphisms and tuberculosis risk.

## 2. Methods

### 2.1. Literature Search

**Figure 1:**
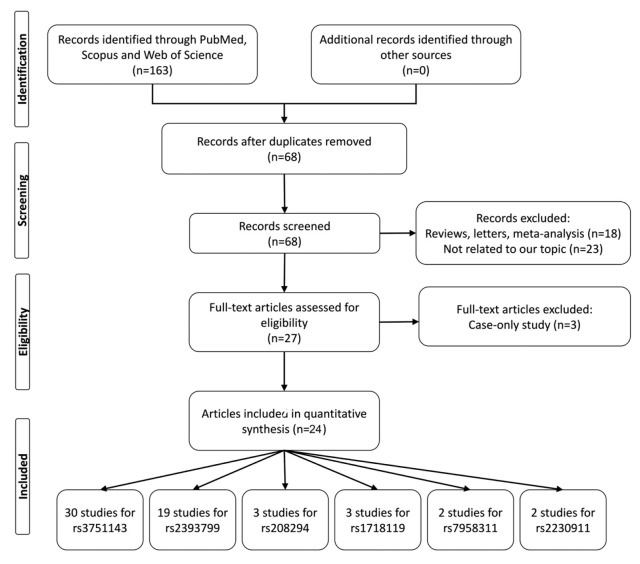
Flow chart illustrates the detailed study selection process of this meta-analysis.

The PubMed, Web of Science, Scopus, and Google scholar databases for all potentially eligible research articles up to November 2018 on the relationship between *P2X7* polymorphisms and TB risk were searched. The search key words used were “*P2X7* or P2X7R” and “tuberculosis” and “polymorphism or variant”. Figure 1 shows the process of recognizing eligible studies. The inclusion criteria were as follows: case-control studies focusing on the association between *P2X7* polymorphisms and TB risk; the frequencies distribution of alleles and genotypes in patients and controls can be extracted. The exclusion criteria were studies that are not associated with *P2X7* polymorphisms and TB risk; overlapping data, conference papers, reviews, meta-analyses; no sufficient data reported.

### 2.2. Data Extraction

Two investigators independently inspected and evaluated the articles for eligibility according to inclusion and exclusion criteria. The following data were recorded from the selected studies such as the first author’s name, publication year, ethnicity, genotyping methods, genotype and allelic profile, as well as the source of controls.

### 2.3. Statistical Analysis

The chi-square test was used to check whether genotypes within the controls conformed to the Hardy-Weinberg equilibrium (HWE). We calculated the pooled odds ratios (ORs) and corresponding 95% confidence intervals (CIs) to assess the association between the *P2X7* polymorphisms and TB susceptibility. The significance of the pooled OR was determined by the Z-test, and a *p*-value less than 0.05 was considered significant. Heterogeneity between the studies was estimated by Q statistic and the I^2^ test. *p* < 0.10 designated significant heterogeneity. If heterogeneity did not exist, a fixed-effects model was used to calculate the pooled ORs; otherwise, a random-effects model was utilized.

Publication bias was inspected with the funnel plot and an asymmetric plot suggests a possible publication bias. Funnel plot asymmetry was further measured using Egger’s linear regression test. *p* value < 0.05 was considered a significant publication bias. Sensitivity analysis was done by neglecting each study in turn to assess the quality and consistency of the results. All statistical analyses were executed using STATA v14.1 software (College Station, TX, USA).

## 3. Results

### 3.1. Study Characteristics

Through a comprehensive literature search and selection based on the inclusion criteria, 59 relevant case-control studies from 24 selected articles [12,13,14,15,16,17,18,19,20,21,22,23,24,25,26,27,28,29,30,31,32,33,34,35] were included in the pooled analysis. There were 30 studies with 5247 cases and 7614 controls on rs3751143 (1513A > C), 19 studies with 3235 cases and 4685 controls on rs2393799 (−762 C > T), 3 studies with 2185 cases and 2107 controls on rs1718119 (Thr348Ala), 3 studies with 1994 cases and 2037 controls on rs208294 (His155Tyr), 2 studies with 2000 cases and 2006 controls on rs7958311, 2 studies with 1853 cases and 1797 controls on rs2230911 included into meta-analysis. The main characteristics of included studies are shown in Table 1.

### 3.2. Main Analysis Results

**Figure 2:**
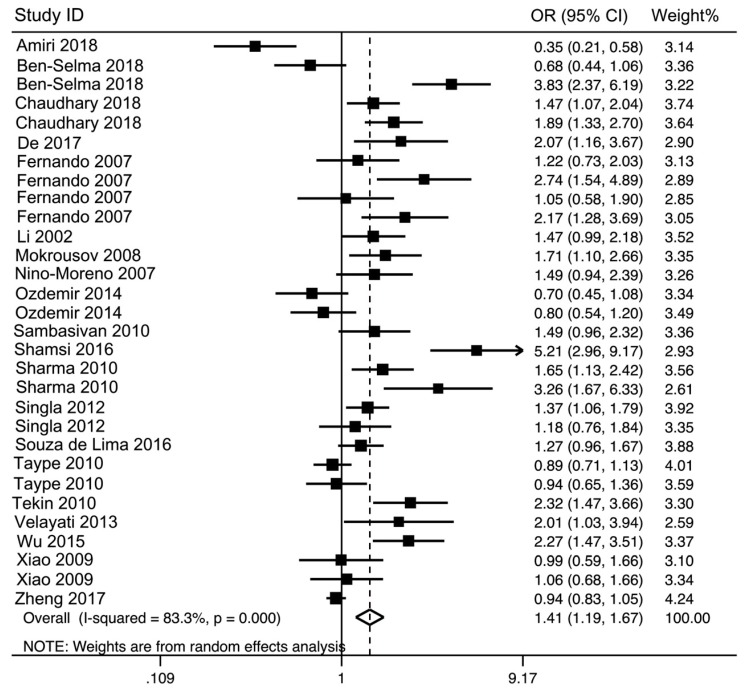
The forest plot for association between P2X7 rs3751143 polymorphism and tuberculosis risk under allelic genetic model (C vs. A).

The Forest plots were applied to show meta-analysis results for each genetic model. Overall, the rs3751143 variant significantly increased the risk of TB in heterozygous codominant (OR = 1.44, 95% CI = 1.17–1.78, *p* = 0.0006, AC vs. AA), homozygous codominant (OR = 1.87, 95% CI = 1.40–2.49, *p* = 0.0004, CC vs. AA), dominant (OR = 1.50, 95% CI = 1.22–1.85, *p* = 0.0002, AC + CC vs. AA), recessive (OR = 1.61, 95% CI = 1.25–2.07, *p* = 0.001, CC vs. AC + AA), and allele (OR = 1.41, 95% CI = 1.19–1.67, *p* < 0.0001, C vs. A) genetic models (Table 2 and Figure 2).

No significant association was found between P2X7 rs2393799, rs1718119, rs208294, rs7958311, and rs2230911 polymorphisms and TB risk (Table 2).

### 3.3. Subgroup Analysis Results

Stratified analysis was achieved (Table 3). The findings proposed that rs3751143 polymorphism increased the risk of pulmonary tuberculosis (PTB) and extrapulmonary tuberculosis (EPTB) in all genetic models. Besides, this polymorphism only contributes to the risk of TB in the Asian population, but not in the Caucasian population (Table 3). The rs2393799 polymorphism was not associated with the risk of TB in the Asian population (Table 3).

### 3.4. Heterogeneity and Publication Bias

In our study, relatively obvious heterogeneities existed under all five genetic models for rs3751143 and rs2393799 (Table 2). For rs1718119, heterogeneities were not observed under all genetic models. For rs208294, heterogeneities were not observed under heterozygous codominant and recessive genetic models. For rs7958311, heterogeneities were not observed under heterozygous codominant and for rs2230911 variant, heterogeneities were not observed under heterozygous codominant and dominant models.

**Figure 3:**
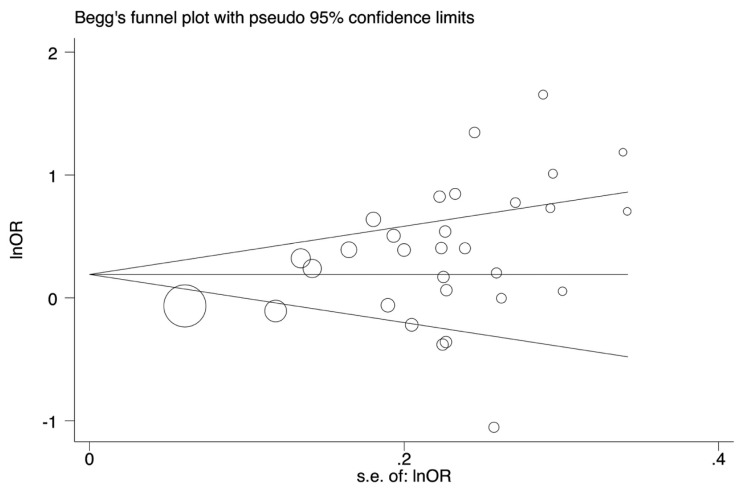
The funnel plot for the test of publication bias. The funnel plot for rs3751143 polymorphism under allele genetic model (C vs. A).

Begg’s tests were done with funnel plot to assess publication bias. Publication bias was found for rs3751143 under five genetic models (Table 2 and Figure 3).

The Begg’s tests indicated no evidence of publication bias for rs2393799, rs1718119, and rs208294 (Table 2) under all genetic models.

### 3.5. Sensitivity Analysis

**Figure 4:**
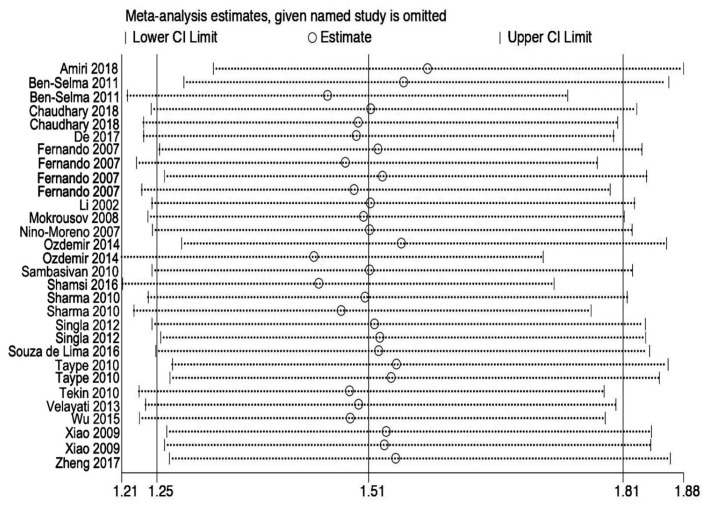
Sensitivity analyses for studies on P2X7 rs3751143 polymorphism and the risk of tuberculosis for C vs. A.

To better inspect the impact of individual study on the pooled OR, we performed sensitivity analysis through deleting each study one by one. Outcomes indicated that ORs were not statistically influenced in all genetic models for rs3751143 (Figure 4), as well as for rs2393799, showing that our results are stable and reliable.

## 4. Discussion

Mounting evidence proposed that host genetic factors are implicated in tuberculosis susceptibility [4,6]. The P2X7R is highly expressed on macrophages and other immune cells [8]. It is a key molecule in the clearance of MTB in macrophages by adenosine triphosphate (ATP)-induced apoptosis of macrophage [8,10]. *P2X7* is polymorphic and several studies investigated the impact of *P2X7* polymorphisms on predisposition to TB [12,13,14,15,16,17,18,19,20,21,22,23,24,25,26,27,28,29,30,31,32,33,34,35]. But these studies failed to reach a consistent conclusion. Therefore, to provide a comprehensive and reliable conclusion, we conducted the present meta- analysis to increase the statistical power of the association. Our findings suggest the *P2X7* rs3751143 (Glu498Ala) polymorphism significantly increased the risk of overall TB. Stratified analysis of this polymorphism significantly increased the risk of PTB and EPTB. Also, the rs3751143 polymorphism increased the risk of TB in the Asian population. Findings did not support an association between rs2393799 (−762 C > T), rs1718119 (Thr348Ala), rs208294 (His155Tyr), rs7958311 (Arg270His), and rs2230911(Thr357Ser) polymorphisms and TB risk.

Ge et al. [36] performed a meta-analysis (*n =* 10) on the association between *P2X7* rs3751143 polymorphism and PTB risk and found that this variant significantly increased the risk of PTB. Another meta-analysis (*n =* 11) performed by Alshammari et al. [37] showed no significant association between rs3751143 polymorphism and risk of TB. Stratified analysis revealed an association between this variant and the risk of TB in the Asian population. The results of a meta-analysis of 8 studies indicated that rs3751143 polymorphism significantly increased the risk of EPTB [38]. Another meta-analysis of 9 studies conducted by Wu et al. [39] revealed that rs3751143 significantly increased the risk of TB. A meta-analysis published by Yi et al. [40] on the association between rs2393799 (−762 C > T) polymorphism and TB susceptibility indicated that this variant is associated with TB risk. Our meta-analysis has more advantages than previous meta-analyses. We included a higher number of relevant published studies. Besides, we evaluated 6 polymorphisms in this meta-analysis.

Several polymorphisms have been described that cause P2X7R loss-of-function (LOF) or gain-of-function (GOF) [8]. The common polymorphism of *P2X7* is rs3751143 (A1513C; Glu498Ala) polymorphism located in exon 13, accountable for LOF. This polymorphism affects the sensitivity of P2X7R to ATP and may contribute to increased susceptibility to MTB infection in humans [13,14,41]. The findings of the present meta-analysis support an association between rs3751143 polymorphism and the risk of TB. Another LOF polymorphism is rs2393799 (−762 C > T), which is located in the promoter of *P2X7* and decrease the expression of P2X7R. The relationship between the rs2393799 polymorphism and susceptibility to TB is still debated [12,13,14,17,18,19,21,23,27,28,29,31,32,33,34], and pooled analysis of all available data did not support an association between this variant and susceptibility to TB. The rs208294 (489 C > T; His155Tyr) is GOF polymorphism. This polymorphism increases the affinity of P2X7R to ATP [42]. Limited studies investigated the impact of this polymorphism on TB susceptibility [14,30,34]. Pooled analysis revealed no evidence of association between this variant and TB risk.

Up until now, only 3 studies investigated the association between rs1718119 (1068 G > A; Thr348Ala) polymorphism and TB risk [30,31,34]. Our findings did not support an association between this polymorphism and TB risk.

*Porphyromonas gingivalis*, a bacterial carcinogen, plays a key role in cancer development by inhibiting apoptosis through several mechanisms. It has been shown that this bacterium secretes an anti-apoptotic enzyme nucleoside diphosphate kinase (NDK) which cleaves ATP and prevents proapoptotic P2X7 receptor activation, consequently modulating ATP/P2X7-signaling pathway [43]. It has been proposed that MTB secrete NDK, which act as a Rho-GTPase-activating protein (Rho-GAP), and covert Guanosine triphosphate (GTP)-bound active form to guanosine diphosphate (GDP)-bound inactive form, eventually facilitating its pathogenesis [44].

Some limitations of our meta-analysis should be acknowledged. Firstly, heterogeneity between studies was evident, which might distort the conclusion of this meta-analysis. Heterogeneity may be partly arising in the differences of ethnicities. Secondly, the sample sizes for some polymorphisms were small. Therefore, the results of this meta-analysis should be interpreted with caution.

Despite these limitations, however, there are still some advantages to having done this meta-analysis. First, this meta-analysis involved more studies than the previous meta-analyses, so the statistical power of our study is higher than the published meta-analysis. Second, we evaluated six polymorphisms of *P2X7*.

## 5. Conclusions

Overall, our meta-analysis proposed that *P2X7* rs3751143 polymorphism may serve as a risk factor for TB in the Asian population. However, further well-designed studies with large sample sizes are necessary to confirm our findings.

## Figures and Tables

**Table 1:** Characteristics of all studies included in the meta-analysis.

First Author	Year	Country	Ethnicity	TB	Source of Control	Genotyping Method	Case/Control	Cases					Controls					HWE (*p*)
rs3751143 A > C								AA	AC	CC	A	C	AA	AC	CC	A	C	
Amiri	2018	Iran	Asian	PTB	PB	PCR-RFLP	100/100	76	21	3	173	27	40	58	2	138	62	<0.001
Ben-Selma	2011	Tunisia	African	PTB	HB	PCR-RFLP	168/150	130	34	4	294	42	104	40	6	248	52	0.395
Ben-Selma	2011	Tunisia	African	EPTB	HB	PCR-RFLP	55/150	19	23	13	61	49	104	40	6	248	52	0.395
Chaudhary	2018	India	Asian	PTB	PB	ARMS-PCR	145/247	63	73	9	199	91	141	95	11	377	117	0.315
Chaudhary	2018	India	Asian	EPTB	PB	ARMS-PCR	100/247	42	42	16	126	74	141	95	11	377	117	0.315
De	2017	India	Asian	PTB	PB	PCR-RFLP	56/60	26	18	12	70	42	36	21	3	93	27	0.978
Fernando	2007	Southeast Asia	Asian	PTB	PB	TaqMan	56/167	34	17	5	85	27	105	55	7	265	69	0.952
Fernando	2007	Southeast Asia	Asian	EPTB	PB	TaqMan	30/167	9	17	4	35	25	105	55	7	265	69	0.952
Fernando	2007	Australia	Caucasian	PTB	PB	TaqMan	49/102	28	21	0	77	21	64	34	4	162	42	0.845
Fernando	2007	Australia	Caucasian	EPTB	PB	TaqMan	50/102	18	28	4	64	36	64	34	4	162	42	0.845
Li	2002	Gambia	African	PTB	HB	PCR-RFLP	325/297	261	58	6	580	70	256	37	4	549	45	0.057
Mokrousov	2008	Russia	Caucasian	PTB	HB	PCR-RFLP	188/126	120	59	9	299	77	96	27	3	219	33	0.511
Nino-Moreno	2007	México	Mixed	PTB	HB	PCR-RFLP	94/110	53	33	8	139	49	70	38	2	178	42	0.215
Ozdemir	2014	Turkey	Asian	PTB	PB	PCR-RFLP	71/160	44	18	9	106	36	76	63	21	215	105	0.176
Ozdemir	2014	Turkey	Asian	EPTB	PB	PCR-RFLP	89/160	47	34	8	128	50	76	63	21	215	105	0.176
Sambasivan	2010	India	Asian	PTB	HB	PCR-RFLP	156/100	89	55	12	233	79	71	21	8	163	37	0.002
Shamsi	2016	Iran	Asian	PTB	HB	PCR-RFLP	100/100	33	66	1	132	68	83	16	1	182	18	0.817
Sharma	2010	India	Asian	PTB	PB	T-ARMS-PCR	181/177	102	75	4	279	83	126	48	3	300	54	0.515
Sharma	2010	India	Asian	EPTB	PB	T-ARMS-PCR	23/177	8	13	2	29	17	126	48	3	300	54	0.515
Singla	2012	India	Asian	PTB	PB	PCR-RFLP	286/392	162	112	12	436	136	258	123	11	639	145	0.420
Singla	2012	India	Asian	EPTB	PB	PCR-RFLP	71/392	45	22	4	112	30	258	123	11	639	145	0.420
Souza de Lima	2016	Brazil	South American	PTB	HB	TaqMan	288/287	170	95	23	435	141	184	89	14	457	117	0.450
Taype	2010	Peru	Caucasian	PTB	HB	PCR-RFLP	498/513	352	130	16	834	162	347	149	17	843	183	0.838
Taype	2010	Peru	Caucasian	EPTB	HB	PCR-RFLP	121/513	82	37	2	201	41	347	149	17	843	183	0.838
Tekin	2010	Turkey	Caucasian	EPTB	HB	PCR-RFLP	74/192	39	28	7	106	42	141	46	5	328	56	0.595
Velayati	2013	Iran	Asian	PTB	HB	PCR- RFLP	79/50	42	35	2	119	39	37	12	1	86	14	0.981
Wu	2015	China	Asian	PTB	PB	PCR-RFLP	103/87	33	49	21	115	91	51	27	9	129	45	0.075
Xiao	2009	China	Asian	PTB	HB	PCR-RFLP	41/384	21	18	2	60	22	221	119	44	561	207	<0.001
Xiao	2009	China	Asian	EPTB	HB	PCR-RFLP	55/384	30	19	6	79	31	221	119	44	561	207	<0.001
Zheng	2017	China	Asian	PTB	PB	TaqMan	1595/1521	972	551	72	2495	695	900	544	77	2344	698	0.655
rs2393799 C > T								CC	CT	TT	C	T	CC	CT	TT	C	T	
Amiri	2018	Iran	Asian	PTB	PB	PCR-RFLP	100/100	8	88	4	104	96	4	95	1	103	97	<0.001
Bahari	2013	Iran	Asian	PTB	PB	ARMS-PCR	150/150	71	54	25	196	104	104	40	6	248	52	0.395
Ben-Selma	2011	Tunisia	African	PTB	HB	ARMS-PCR	168/150	16	57	95	89	247	14	51	85	79	221	0.130
Ben-Selma	2011	Tunisia	African	EPTB	HB	ARMS-PCR	55/150	4	15	36	23	87	14	51	85	79	221	0.130
Chaudhary	2018	India	Asian	PTB	PB	ARMS-PCR	145/247	62	67	16	191	99	101	111	35	313	181	0.614
Chaudhary	2018	India	Asian	EPTB	PB	ARMS-PCR	100/247	44	48	8	136	64	101	111	35	313	181	0.614
Li	2002	Gambia	African	PTB	HB	PCR-RFLP	323/347	23	118	182	164	482	44	140	163	228	466	0.111
Mokrousov	2008	Russia	Caucasian	PTB	HB	ARMS	190/127	86	87	17	259	121	65	46	16	176	78	0.093
Nino-Moreno	2007	México	Mixed	PTB	HB	ARMS	92/110	8	32	52	48	136	15	44	51	74	146	0.275
Sambasivan	2010	India	Asian	PTB	HB	PCR-RFLP	156/100	38	88	30	164	148	15	49	36	79	121	0.801
Shamsi	2016	Iran	Asian	PTB	HB	PCR-RFLP	100/100	1	99	0	101	99	6	93	1	105	95	<0.001
Singla	2012	India	Asian	PTB	PB	ARMS	286/392	143	115	28	401	171	231	143	18	605	179	0.485
Singla	2012	India	Asian	EPTB	PB	ARMS	71/392	40	25	6	105	37	231	143	18	605	179	0.485
Songane	2012	Indonesia	Asian	PTB	PB	MassARRAY	842/844	181	413	248	775	909	177	412	255	766	922	<0.001
Velayati	2013	Iran	Asian	PTB	HB	ARMS	79/50	10	67	2	87	71	3	47	0	53	47	<0.001
Wu	2015	China	Asian	PTB	PB	PCR-RFLP	103/87	35	47	21	117	89	9	30	48	48	126	0.202
Xiao	2009	China	Asian	PTB	HB	ARMS	38/384	23	11	4	57	19	208	135	41	551	217	0.009
Xiao	2009	China	Asian	EPTB	HB	ARMS	58/384	40	12	6	92	24	208	135	41	551	217	0.009
Zhou	2018	China	Asain	EPTB	HB	Mass Spectrometry	179/324	81	77	21	239	119	122	143	59	387	261	0.137
rs1718119 G > A								GG	AG	AA	G	A	GG	AG	AA	G	A	
Bahari	2013	Iran	Asian	PTB	PB	T-ARMS-PCR	150/150	63	72	15	198	102	66	69	15	201	99	0.622
Zheng	2017	China	Asian	PTB	PB	TaqMan	1568/1454	1090	440	38	2620	516	978	417	59	2373	535	0.087
Zhu	2016	China	Asian	PTB	HB	MassARRAY	467/503	372	91	4	835	99	412	89	2	913	93	0.222
rs208294 G > A								GG	AG	AA	G	A	GG	AG	AA	G	A	
Chaudhary	2018	India	Asian	Mixed	PB	PCR-RFLP	245/246	56	147	42	259	231	49	143	54	241	251	0.011
Zheng	2017	China	Asian	PTB	PB	TaqMan	1570/1467	597	732	241	1926	1214	578	679	210	1835	1099	0.642
Zhou	2018	China	Asian	EPTB	HB	Mass Spectrometry	179/324	22	80	77	124	234	70	145	109	285	363	0.099
rs7958311 G > A								GG	AG	AA	G	A	GG	AG	AA	G	A	
Zheng	2017	China	Asian	PTB	PB	TaqMan	1533/1503	402	797	334	1601	1465	396	775	332	1567	1439	0.199
Zhu	2016	China	Asian	PTB	HB	MassARRAY	467/503	114	215	138	443	491	137	262	104	536	470	0.300
rs2230911 C > G								CC	CG	GG	C	G	CC	CG	GG	C	G	
Souza de Lima	2016	Brazil	South American	PTB	HB	TaqMan	288/288	170	95	23	435	141	193	89	6	475	101	0.245
Zheng	2017	China	Asian	PTB	PB	TaqMan	1565/1509	1029	482	54	2540	590	997	467	45	2461	557	0.274

List of abbreviations: PTB: Pulmonary Tuberculosis; TB: Tuberculosis; EPTB: Extrapulmonary Tuberculosis; PCR-RFLP: PCR-Restriction fragment length polymorphism; ARMS-PCR: Amplification-refractory mutation system-PCR; TaqMan: probes used in quantitative PCR; T-ARMS-PCR: Multiplex Tetra-Primer Amplification Refractory Mutation System-PCR; MassARRAY: Non-fluorescent detection platform utilizing mass spectrometry to accurately measure PCR-derived amplicons.

**Table 2:** The pooled ORs and 95% CIs for the association between *P2X7* polymorphisms and tuberculosis susceptibility.

Polymorphism	No.	Genetic Model	Association Test			Heterogeneity			Publication Bias Tests	
			OR (95%CI)	Z	P	χ2	I^2^ (%)	P	Egger’s Test *p*-Value	Begg’s Test *p*-Value
rs3751143	30	AC vs. AA	1.44 (1.17–1.78)	3.42	0.0006	158.86	81.7	0.000	0.016	0.016
		CC vs. AA	1.87 (1.40–2.49)	4.26	0.0004	61.79	54.7	0.000	0.002	0.047
		AC + CC vs. AA	1.50 (1.22–1.85)	3.78	0.0002	178.85	83.8	0.000	0.007	0.018
		CC vs. AC + AA	1.61 (1.25–2.07)	3.65	0.001	50.79	44.9	0.005	0.006	0.051
		C vs. A	1.41 (1.19–1.67)	3.97	<0.0001	173.41	83.3	0.000	0.006	0.066
rs2393799	19	CT v CC	1.00 (0.83–1.20)	0.01	0.989	32.34	44.3	0.020	0.460	0.753
		TT vs. CC	0.99 (0.68–1.44)	0.04	0.965	73.55	75.5	0.000	0.935	0.510
		CT + TT vs. CC	0.97 (0.77–1.23)	0.22	0.825	58.41	69.2	0.000	0.557	0.649
		TT vs. CT + CC	0.99 (0.74–1.32)	0.08	0.938	71.34	74.8	0.000	0.962	0.680
		T vs. C	0.98 (0.83–1.17)	0.20	0.844	95.15	81.1	0.000	0.657	0.763
rs1718119	3	AG vs. GG	0.99 (0.86–1.13)	0.16	0.88	1.13	0	0.57	0.308	0.602
		AA vs. GG	0.70 (0.49–0.99)	1.99	0.05	3.56	44	0.17	0.136	0.117
		AG + AA vs. GG	0.96 (0.84–1.09)	0.68	0.50	2.22	10	0.33	0.312	0.602
		AA vs. AG + GG	0.70 (0.49–1.00)	1.98	0.05	3.24	38	0.20	0.141	0.117
		G vs. A	0.93 (0.83–1.04)	1.24	0.21	3.49	43	0.17	0.242	0.602
rs208294	3	AG vs. GG	1.03 (0.93–1.23)	0.89	0.37	3.77	47	0.15	0.694	0.602
		AA vs. GG	1.18 (0.69–2.02)	0.61	0.54	8.99	78	0.010	0.900	0.602
		AG + AA vs. GG	1.16 (0.80–1.68)	0.76	0.45	6.50	69	0.04	0.751	0.602
		AA vs. AG + GG	1.08 (0.78–1.49)	0.47	0.64	5.60	64	0.06	0.904	0.602
		A vs. G	1.09 (0.85–1.40)	0.70	0.49	8.82	77	0.01	0.860	0.602
rs7958311	2	AG vs. GG	1.01 (0.87–1.17)	0.09	0.93	0.02	0	0.88		
		AA vs. GG	1.23 (0.77–1.95)	0.87	0.38	5.15	81	0.02		
		AG + AA vs. GG	0.81 (0.50–1.31)	0.87	0.38	8.09	88	0.004		
		AA vs. AG + GG	1.24 (0.76–2.01)	0.87	0.38	8.09	88	0.004		
		A vs. G	1.11 (0.88–1.40)	0.87	0.38	5.18	81	0.02		
rs2230911	2	CG vs. CC	1.03 (0.90–1.19)	0.42	0.67	0.95	0	0.33		
		GG vs. CC	2.10 (0.58–7.66)	1.13	0.26	6.65	85	0.010		
		CG + GG vs. CC	1.01 (0.88–1.16)	0.13	0.89	0.28	0	0.60		
		GG vs. CG + CC	2.03 (0.60–6.94)	1.13	0.26	6.12	84	0.01		
		G vs. C	1.22 (0.83–1.80)	1.03	0.30	6.09	84	0.01		

List of Abbreviations: OR: Odds Ratio; CI: Confidence interval Z: Z-score; P: Probability; χ2: χ2 test; I^2^: I^2^ value.

**Table 3:** Stratified analysis of P2X7 polymorphisms and tuberculosis risk.

Parameters	No.	AC vs. AA		CC vs. AA		AC + CC vs. AA		CC vs. AC + AA		C vs. A	
		OR (95% CI)	*P*	OR (95%CI)	*P*	OR (95%CI)	*P*	OR (95%CI)	*P*	OR (95%CI)	*P*
rs3751143											
Tuberculosis											
PTB	21	1.35 (1.05–1.74)	0.020	1.50 (1.10–2.04)	0.010	1.39 (1.09–1.78)	0.009	1.34 (1.04–1.73)	0.020	1.31 (1.09–1.58)	0.004
EPTB	9	1.68 (1.17–2.42)	0.005	2.62 (1.19–5.78)	0.020	1.84 (1.21–2.79)	0.004	2.05 (1.07–3.93)	0.030	1.67 (1.16–2.42)	0.006
Ethnicities											
Asian	19	1.48 (1.09–2.00)	0.010	1.70 (1.17–2.48)	0.006	1.53 (1.13–2.06)	0.006	1.47 (1.07–2.00)	0.020	1.57 (1.22–2.02)	0.0005
Caucasian	6	1.47 (0.99–2.17)	0.05	1.56 (0.70 − 3.51)	0.28	1.49 (0.98–2.26)	0.06	1.36 (0.70–2.66)	0.37	1.37 (0.96–1.97)	0.09
African	3	1.45 (0.66–3.19)	0.36	2.16 (0.33–13.95)	0.42	1.60 (0.62–4.13)	0.33	1.89 (0.41–8.81)	0.42	1.56 (0.62–3.94)	0.35
rs2393799		CT vs. CC		TT vs. CC		CT + TT vs. CC		TT vs. CT + CC		C vs. T	
Asian	14	0.92 (0.74–1.14)	0.44	0.87 (0.54–1.41)	0.58	0.86 (0.66–1.14)	0.30	0.92 (0.61–1.40)	0.70	0.98 (0.83–1.17)	0.84
